# Probability landscapes for integrative genomics

**DOI:** 10.1186/1742-4682-5-9

**Published:** 2008-05-20

**Authors:** Annick Lesne, Arndt Benecke

**Affiliations:** 1 Institut des Hautes Études Scientifiques, Bures sur Yvette, France; 2 Institut de Recherche Interdisciplinaire – CNRS USR3078 – Université Lille I, France

## Abstract

**Background:**

The comprehension of the gene regulatory code in eukaryotes is one of the major challenges of systems biology, and is a requirement for the development of novel therapeutic strategies for multifactorial diseases. Its bi-fold degeneration precludes brute force and statistical approaches based on the genomic sequence alone. Rather, recursive integration of systematic, whole-genome experimental data with advanced statistical regulatory sequence predictions needs to be developed. Such experimental approaches as well as the prediction tools are only starting to become available and increasing numbers of genome sequences and empirical sequence annotations are under continual discovery-driven change. Furthermore, given the complexity of the question, a decade(s) long multi-laboratory effort needs to be envisioned. These constraints need to be considered in the creation of a framework that can pave a road to successful comprehension of the gene regulatory code.

**Results:**

We introduce here a concept for such a framework, based entirely on systematic annotation in terms of probability profiles of genomic sequence using any type of relevant experimental and theoretical information and subsequent cross-correlation analysis in hypothesis-driven model building and testing.

**Conclusion:**

Probability landscapes, which include as reference set the probabilistic representation of the genomic sequence, can be used efficiently to discover and analyze correlations amongst initially heterogeneous and un-relatable descriptions and genome-wide measurements. Furthermore, this structure is usable as a support for automatically generating and testing hypotheses for alternative gene regulatory grammars and the evaluation of those through statistical analysis of the high-dimensional correlations between genomic sequence, sequence annotations, and experimental data. Finally, this structure provides a concrete and tangible basis for attempting to formulate a mathematical description of gene regulation in eukaryotes on a genome-wide scale.

## Background

The approximately 6,000 to 100,000 genes encoded in different eukaryotic genomes display complex patterns of activity according to the physiological state of the cell and the organism [1]. The resulting cell and cell-state specific transcriptome profiles result from a combination of tightly controlled regulatory events in response to intra-, extra-, and inter-cellular signals [2]. These transcription programs are blurred by different stochastic influences, however, they define the cellular state and activity [3-5]. Almost all known disorders including cancer, genetic syndromes, and pathogen induced diseases are characterized by altered transcriptome profiles [2,6]. Often the molecular basis for pathology is found in affected gene regulatory signaling [6]. Understanding gene regulation therefore required not only for comprehending an organism's physiology but also for developing novel strategies for interference with physiopathology [1,2,6,7]. Since the discovery of DNA as the carrier of genetic information, much progress has been made in the experimental identification of protein coding sequences. Since the genetic code has been elucidated such sequences can be predicted with relatively high fidelity. On the other hand, non-protein coding genes and especially small RNAs are much harder to identify on the basis of sequence information alone [8]. Even more challengingly, many attempts are currently being made to improve the predictive power of sequence statistics for regulatory processes, but we are only just beginning to understand the sequence structures of regulatory sites [3,9,10]. In view of the fact that all the protein-coding genes in eukaryotes in toto make up as little as two percent of the entire genomic sequence we are far from having an understanding of the genome [2]. The vast majority of the eukaryotic genome is involved in various, often non-understood processes such as sequence buffering or evolutionary experimentation, but most importantly in the control of gene regulation [1,2]. Gene regulatory control has been a focus of attention since the 1970s because it is the key to understanding the intricate interplay among genes under various physiological and pathological conditions. [11]. Numerous insights have been gained into the identity and function of individual transcription regulatory molecules, as well as the regulatory sequences to which they bind [12]. However, today only about three hundred transcription factors with an average of about twenty regulatory sequence elements have been well characterized experimentally for *e.g*. the human genome [13]. It is estimated, however, that the human genome encodes some 3,000 sequence specific transcription factors and at least 100,000 regulatory elements [2,12,13]. Despite this enormous discrepancy, five fundamental properties of gene regulatory coding have been established [1]. First, the gene regulatory code is bi-fold degenerate. Hence, and in striking contrast to the genetic code, even a complete knowledge of all transcription regulatory molecules and all regulatory sequence elements would not allow those elements to be mapped unequivocally in the absence of further information. Second, the gene regulatory code is interpreted in a context-dependent manner by the cellular machinery. Depending on either the sequence environment or the physiological environment the very same regulatory element has drastically different regulatory activities. Third, the gene regulatory code is combinatorial. Any regulatory signal in eukaryotes is conveyed by at least three but up to more than ten sequence specific DNA binding activities. The individual contributions of those regulatory factors act synergistically such that the activity AB ≠ A+B and even AB ≠ BA. Fourth, the gene regulatory code is distributed. Regulatory sequence elements are often found hundreds of kilobases away from the site of gene transcription initiation, are non-continuous, and are sometimes even shared among different genes. And finally, the gene regulatory code is composed of DNA sequence and DNA-associated protein sequence elements. During the past two decades increasing evidence has accumulated that covalent post-translational modifications to DNA-associated proteins contribute significantly to the design and properties of the gene regulatory code. Here especially the histone and non-histone nucleosomal proteins play a major role [2]. The eukaryotic genome is at any moment in time tightly packed into the chromatin structure, with histone-containing nucleosomes being the fundamental building block [2,14]. About one nucleosome is associated with every 160–200 basepairs of DNA, and participates in the regulation of gene activity by influencing for example access to regulatory DNA sequences [2,14]. On the basis of these observations a histone- or chromatin-code hypothesis has been developed that places chromatin at the heart of gene regulatory control [1,2,15].

Therefore, the gene regulatory code and its cellular interpretation entail multilevel, distributed, context- and history-dependent information processing [1,2,15]. These facts, taken individually or together, preclude any brute force statistical approach to breaking the gene regulatory code. Likewise, given the sheer size of a eukaryotic genome and the impracticality of fully exploring the sequence space using mutagenesis and subsequent phenotypical analysis, a brute force experimental approach is also excluded. Only a combination of advanced statistical analysis with high-throughput whole-genome experimental data might pave the way to deciphering the regulatory code. This assertion is today widely acknowledged in the literature and different research programs have emerged that try to achieve such an integrated analysis [16-19]. Such approaches are challenged by different constraints. The increasingly available genomic sequences are still not finalized as different regions of the eukaryotic genome are difficult to sequence or assemble. More importantly, as many genes, especially non-protein coding genes, still need to be identified [8], the sequence annotations of eukaryotic genomes are under continual discovery-driven change. Experimental methods for analyzing DNA-based events on a genome-wide scale and in a high-throughput manner are not only very expensive but also just in their infancy in terms of sensitivity, robustness, and coverage [20,21]. Methods for measuring the same biological process or object are often heterogeneous in their technical design and in the absence of independent standards and controls lead to similarly heterogeneous data. Many exciting and urgently required new technologies are on the horizon, such as massive parallel sequencing, but are still far from routine use in the laboratory. Finally, the combinatorial complexity of the question (10^5 ^genes making up at least a thousand distinct genetic programs in some 10^12^–10^14 ^individual cells of a typical higher eukaryote), requires multi-laboratory and probably decades-long coordinated efforts. Any framework for achieving integrated experimental and sequence statistical analysis must therefore not only be systematic and coherent, but also portable and evolvable to accommodate future advances in genome biology. The challenge here can be compared to the development of open-source, portable, and extendable digital data formats for the long-term storage of information, which is currently a major concern for the computer science community [22], and will need to be combined with a similar open, portable, and extendable set of analysis tools. We present here a concept for such a framework. We show how any type of existing and future experimental data, theoretical predictions and models, as well as sequence information may be coherently integrated. The proposed strategy thereby satisfies all the above criteria.

## Results

### Genome probability landscapes

The different genome sequences at our disposal today are characterized by several important limitations: (i) they are average sequences obtained by sequencing several (not necessarily many) individuals that may not be representative and may differ from one another [23,24]; (ii) they contain gaps of regions that are either resistant to the sequencing chemistry or simply not present in a significant sub-population in the sequenced individuals [23,24]. Those gaps are of various or unknown length. (iii) In some cases two or more bases occur with similar frequency in the sequenced individuals, and averaging does not produce an unambiguous result. Those positions are often indicated simply by an 'N' in the linear sequence [23,24]. (iv) Genome sequences from different sources for the same organism may differ [23,24]; (v) true errors in the sequence and wrong sequence concatenation are still quite frequent [23,24]. The currently used format for representing genomic sequences is a letter code that mostly does not indicate of the location of gaps. On average, dozens of new genome releases with ever increasing quality are published during the course of a year.

Owing to ever-increasing sequence throughput together with a decrease in cost per base-pair, we can very soon expect to see genome sequences that take account the base frequency at each position through the concurrent sequencing of many representative individuals [25]. As there is significant non-random variation in the occurrence rate of a given nucleotide at some positions, as well as non-negligible random variation at other positions, we will for the first time obtain a glimpse of the sequence variability on a genome-wide scale. Such represented genomes will thus contain information on *e.g*. single nucleotide polymorphisms (SNPs) [25].

In the long term future one can also expect that it will become feasible to sequence a large number of individuals of a given species separately [25]. Individual sequences then can be compared, clustered into sub-populations, and analyzed for correlations in the base frequencies at given positions. Such genomic sequences would thus also contain complete information on *e.g*. haplotype variation between sub-populations and region copy number [25,26].

**Figure 1:**
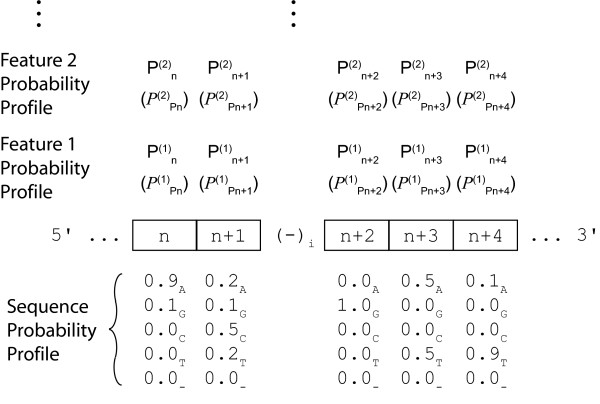
**The principle of genome probability profiles**. Annotation of genome sequence probability profiles with feature probability profiles.

Formalisms for systematic gene regulatory research have to be able to accommodate today's genome sequence representations as well as possible future formats. Furthermore, new releases in any given format have to be handled. For the former, a solution adapted to frequency distribution representations is used. Most importantly, treating genomes as nucleotide frequency distributions is equivalent to casting a genome as a probability profile. We argue that for efficient integration of experimental or theoretical data (hereafter also referred to as *features*) from heterogeneous sources and their correlation with sequence statistics all information has to be converted into similar nucleotide-based probability profiles. The entire problem is thus converted into a homogeneous genome probability multilayer landscape in which any individual feature is annotated using a separate profile. Furthermore, as the quality of the observation or prediction at each nucleotide does vary, a second measure is provided, amounting to a probability density defining the quality of the initial probability value, to capture this inhomogeneity (Figure 1). In the following paragraphs we discuss how this can be achieved. The resulting structure can be used to apply Rényi entropy-based high-dimensional correlation functions for efficient hypothesis testing in the context of gene regulatory control.

### Sequence annotations

Sequence annotations, even more than the genomic sequence itself, undergo frequent revisions. Many genes remain to be identified or confirmed experimentally in various eukaryotic genomes. As discussed in the introduction, this is especially true for small RNA coding genes where research is still at a very early stage [8]. In order to map gene-bound experimental data correctly to the genome sequence one has to use gene annotation information. Furthermore, gene-transcript based experimental data must first be mapped to a gene annotation and then subsequently to the genomic sequence. As a single gene can produce a multitude of different transcripts through alternative splicing, alternate promoter usage and other biological processes, this two-level mapping is a challenge in itself [27,28]. When considering proteomics data the problem is less complicated in principal as the expressed protein information can either be mapped directly back to the genomic sequence using so called proteogenomic mapping or be mapped to transcript information and then via gene information to the genomic sequence. Again, owing to post-translational modifications and processing and the degeneracy of the genetic code, this is far from trivial and often not possible to achieve unequivocal. Therefore, a probability based annotation approach almost imposes itself.

**Figure 2:**
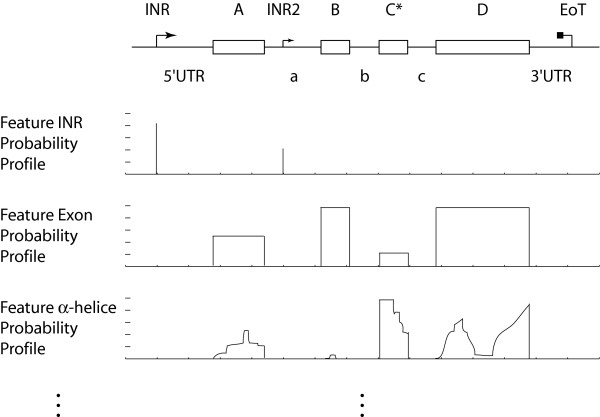
**Generating feature probability profiles from gene and gene transcript annotations**. INR: initiator region (transcription start-site); INR2: alternate transcription start-site; EoT: end of transcript; {A, B, C, D}: exon; C*: alternative spliced exon; UTR: untranslated region; {a, b, c}: intron.

Many different features characterize a gene within the genome. The initiation region with the first translated nucleotide (INR), the exon-intron structure, 5' and 3' untranslated regions (UTR), and also information on the structure and stability of its transcript, or a possible protein translated from the transcript, can be taken into consideration [29]. For many of those features we still do not have a very good picture on a genome-wide scale. However, for sake of future hypothesis testing, the formalism of sequence annotation should be able to account consistently for any possible feature one might choose in the future. We again think that this is best achieved by using probabilities. This contention is further supported by the observation that foregoing features are neither necessarily present nor necessarily unique; for instance, alternate promoter usage often also leads to alternate transcription start-site selection, or alternative splicing to the presence or absence of a exon sequence in the transcript. As shown in Figure 2 such information can be translated into probability profiles along the genome, and can be readily generated from existing sequence annotation databases [30-32]. In order to account for varying levels of quality those annotation data should also be associated with a quality probability (Figure 1). The need to create probability profiles for gene features is more readily appreciated when the different experimental data and their structure are considered in relation to these sequence annotations.

### Experimental data

Although there are problems associated with their heterogeneity in design, scope, exhaustiveness, and quality, or between different technologies, two main issues need to be addressed with respect to experimental whole-genome data. First, the nature of the data is drastically different from one data source to another. Some directly concern the DNA structure itself, others such as protein levels apply to the DNA sequence only indirectly. Both have to be treated separately to begin with and then integrated into a single coherent formalism. The other concern is that most functional genomics data do not provide absolute quantification of the objects under study but rather relative quantities between different objects and even more often for a single object between two different experimental conditions. Therefore, inter-assay normalization and standardization has to be resolved [33].

#### Nature of experimental data

Despite sequence information, functional genomics today creates data for gene expression (transcriptomics), protein expression (proteomics), comparative genome-region amplification/loss (CGH), single nucleotide polymorphism (SNP), chromatin and chromatin factor DNA association (ChIP-on-chip), chromatin domains (*e.g*. telo-/centromeres, PEV, MAR), haplotype mapping, cytosine methylation status, chromosomal aberrations, spatial chromosome and chromosome domain localization [34]. It is likely that many others, such as high resolution mutation analysis, chromatin fiber structure and dynamics analysis, or local sub-nuclear ionic strength measurements coupled to chromatin domain sub-nuclear localization will be developed in the future. These methods have drastically different resolution ranging from single nucleotide (SNP, cytosine methylation) to entire chromosomes (10^8 ^nucleotides, spatial chromosome localization) [34]. To integrate such data coherently they have to be remapped to the single nucleotide level. Furthermore, as experimental data only represent snapshots of a dynamic molecular reality in the cell, and because these snapshots are further biased through the technology itself, combined with the fact that they are often generated under non-identical conditions, and finally also possess varying time resolution, they need to be translated into probabilities for events or objects to occur. Thereby the same probabilities and the corresponding quality measures for lower-resolution experiments are simply attributed to all the nucleotides in the region concerned, as in the case of gene feature annotation (Figure 2). The resulting probability profiles can then be co-analyzed regardless of the resolution and quality of the contributing data. Only by using such a systematic and coherent approach to data annotation can the genomic sequence questions of whether for instance a given cytosine methylation event correlates with the chromatin fibre dynamics in a given spatial chromosome location be addressed.

#### Data normalization

The problem of normalization between experimental data generated using different technologies or under different experimental conditions vanishes if probabilities are used. Translating experimental data into probabilities is not trivial but can be achieved in the following manner. Again the nucleotide resolution of the technology separates two cases. SNP and similar single nucleotide resolution data can be interpreted, similarly to the sequence data themselves, as frequency distributions. The quality measure for each probability at a given nucleotide thereby directly reflects the confidence that the true frequency distribution has been faithfully represented, and can be determined by standard statistics on basis of the concrete data (see paragraph 3).

In the second case, for lower resolution at the genomic sequence level, and comparative technologies that do not provide absolute object/process quantification, several considerations become pertinent. We discuss them here for sake of clarity in detail only for the example of transcriptome data; however, they apply similarly to any type of experimental setup falling into this second category.

Transcriptome profiles are thought to provide a measure for the expression level, or expression-level change between two experimental conditions, of a large number of gene transcripts simultaneously [20]. Currently, the main limitations of these transcriptome profiles are: (1) no absolute quantification, (2) no complete reference data-sets available, (3) probes or probe-sets do not cover the entire transcript length, (4) probes are not isoform specific, (5) known and unknown probe cross-reactivity, and (6) relative low precision [20,28,34].

No absolute quantification of transcripts can be achieved because on the one hand no satisfactory physico-chemical models for the hybridization of two nucleic acids exist. As such, differences between probe and target sequences between individual probe-target sets, which lead to distinct hybridization kinetics for such sets, can neither be analyzed for absolute quantification nor be normalized amongst each other. This could partially be overcome if complete reference datasets were available. Such a reference dataset would be a catalogue of all probe-target signal intensities in all available physiological cell types and tissues. In consequence the reference dataset then provides a reference signal under physiological condition to which any experimental biological sample intensity could be compared. Since not all tissues have been well identified and characterized such a reference dataset is still far from availability. However, significant efforts are being made in this direction [35]. Until those efforts have been completed, signal intensities obtained for a given probe-target set are an unknown nonlinear function of absolute target concentration, and comparable probe-target intensities for two different sets do not necessarily reflect similar target concentrations. Therefore, only probe-target signals for the very same probe-target set can be directly compared between different experimental conditions. This is similarly true for other high-throughput functional genomics technologies such as proteomics approaches [34]. While one can expect that ever better physico-chemical models for the hybridization process will emerge [36] and in the future contribute to solving the problem of non-absolute quantification, any attempt to couple such experimental data with genomic sequences today needs to account for this insufficiency. The way to achieve this is by defining a probability of maximal signal-intensity individually for every probe-target sequence. This probability is rescaled whenever new experimental data indicate that under different experimental conditions a given probe-target set can generate an even higher signal intensity within the dynamic range of the technology such that the highest signal intensity ever observed for a given probe is the unity probability event (see paragraph 3).

The reasons for alternate transcripts from a single gene have been addressed briefly above. Because knowledge of the mechanisms leading to alternate transcripts and the sequences concerned in such processes is incomplete, one can not systematically predict where probes need to be placed to discriminate the occurrence of alternate transcripts [20,34]. Furthermore, for technical reasons it is not yet possible to construct probe-sets for a single gene that would cover any possible combination of alternate transcripts as the combinatorics of the problem simply lead to too high numbers [20,34]. Again, much effort is currently being devoted to achieving complete transcript coverage for some model organisms. However, even optimistic estimates indicate that it will take another several years before such isoform-specific arrays become available. Today's strategies in probe design are directed towards probe sets covering as many alternate transcripts as possible without being able to distinguish between them [28]. Therefore probe sets are often found in the 3' region of genes, which are assumed to be less variable then the 5' regions and therefore common to more alternate transcripts. Annotations of signal intensities on a genomic sequence need to take this particular probe design into account. As a general rule the measured signal intensity for a given probe should only be directly annotated to the very same nucleotide sequence in the genome. In most cases the probe intensity measure can be assumed to reflect the relative abundance of the entire targeted exon; however, the identical abundance estimate should not necessarily or automatically be assigned to other non-covered exons. For genes covered with a single probe-set this strategy does not create any difficulty for downstream correlation analysis. However, it has to be kept in mind that the gene activity estimate might be severely biased as for instance the existence of yet undiscovered alternate transcripts participating in the signal estimate, or not being covered by the probe-set, is not deducible from the data [28]. Therefore, the validity of the estimation can not be self-consistently assessed.

Whenever several probe-sets are available to a single gene, the data are likely to be of better quality; however, their interpretation is more challenging. It is estimated today that every gene in a higher eukaryote generates on average four alternate transcripts [37]. Examples of genes are known that generate many times this number of alternate transcripts [37]. Moreover, the contribution to the signal estimate of transcripts unrelated to the gene against which the probe was designed is completely unknown. Furthermore, the same problem of non-absolute quantification and hence incomparability of the different probe signal intensities applies when comparing two different probes for a single gene as much as when comparing two different genes [33]. As no systematic integration of the different probe signal intensities can be proposed, the following strategy should be employed: Every individual probe is considered to measure a distinct object. Correlations (see below) are then calculated as if the different probes designed to quantify a single gene were quantifying individual genes. Cross-correlation analysis over large, many-condition datasets will over time uncover correlations between probes of very different genes indicating cross-hybridization. Such information then can be used to improve the transcript-to-probe annotation [27,28]. Similar conclusions can be drawn for the other technologies that produce average signals over many nucleotides. As a matter of fact, only whole genome tiling arrays with high redundancy (*e.g*. overlap of adjacent sequence probes) would overcome some of the problems posed here [34].

### Probability landscapes as a common denominator

**Figure 3:**
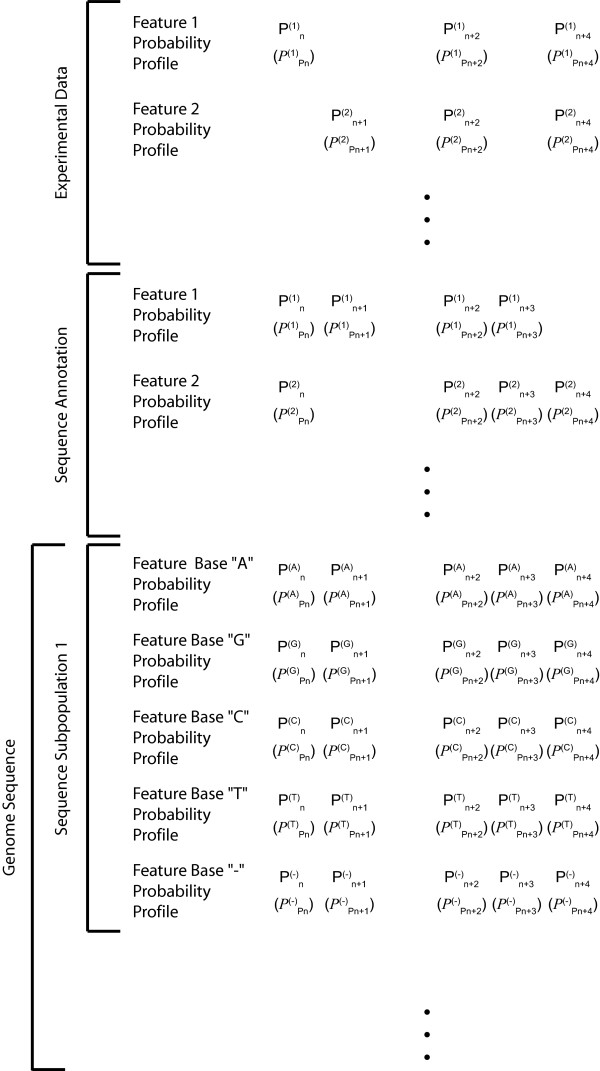
**Genomic probability landscapes – unified structures for genomic analysis**. Genomic sequence information, empirical sequence annotations and whole genome experimental data are converted into probability profiles along the genome primary sequence. Every profile consists of a primary probability for the feature at the given position and a secondary probability capturing the quality of the feature at the same position. New information can either be used to replace existing probability layers or added as new layer. The ensemble of information creates a probability landscape. Rescaling of probabilities can be easily achieved by vertical integration of the data base information.

We have discussed above three distinct types of information, (i) genomic sequence information, (ii) sequence annotation information, and (iii) systematic genome-wide experimental data. We have argued that in order to integrate these different types of information for co-analysis they need to be transformed into frequency distributions along the genome sequence, which is itself represented by a probability distribution (Figure 3).

The proposed probability landscapes are the only systematic and coherent way of handling the existing various and heterogeneous information and any kind of future information that might become available without putting any constraints or bias on its nature. Importantly, the probability layers will contain gaps where no information is available. Those should not be confused with sequences where the probability, of *e.g*. gene expression, is zero. We speak here of globally non-continuous profiles, which are nevertheless locally continuous. As can be seen, a side effect of those gaps is to render cross-correlation analysis more efficient. The proposed structure is homogenous as any information is translated to probability layers. The structure is easily updatable, as either probability layer can be replaced with improved or more accurate information. Both elements of a given layer, nucleotide feature probabilities and probabilities of nucleotide feature probabilities, can be rescaled according to new information. And finally, additional feature probability layers can be added at will in tune with novel technological or theoretical advances. Taken together, the structure and the quality of any information can easily evolve in tune with novel discovery-driven insights and technical developments. The entire landscape needs to be recalculated with every new genome release, as argued above, as those might change absolute position information. The requirement for recalculation of the entire landscape actually is not so much a technical limitation, but rather renders explicit the notion of local sequence-bound information across all layers with long-range or global consequences for biological information processing. However, this process is straightforward and can be automated, making it as much efficient as it is portable. A more detailed description of the constructive procedures is given in the methods section.

## Discussion

We have sketched here a unified structure consisting of probabilities and associated quality estimates – in the form of probability densities – to integrate any type of relevant genomic information into a coherent annotation. Most importantly, we show that the genomic sequence itself, its annotation with empirically derived features, and any type of functional genomics data can be described in this manner. The rationale of this probabilistic description is not necessarily to account for an underlying stochasticity, though for some biological processes this is indeed utilized, but rather to provide an efficient way to formulate partial knowledge and turn relative data of very heterogeneous nature and origin into absolute values and a homogeneous representation of the initial observations. Genome probability landscapes are systematic as any type of relevant information can be correctly and sensibly projected upon sequence distributions. This projection has a single nucleotide resolution, producing a (at least locally) continuous profile. The proposed framework is coherent, as any information is converted without exception into the very same structure: probabilities with associated probability densities for local quality estimation. While the proposed representation of information is far from optimal in terms of compression, it provides a direct, systematic, and coherent interface for analysis, thus rendering analytical calculation extremely efficient. The systematic nature of genome probability landscapes and their coherent structure allows easy exchange of information between different research teams. The simple structure of the resulting data also makes the framework easily portable between different computing environments as there is no real need for a solid database structure to generate, store, and handle the information. Finally, as any type of future information can also be included in the very same manner into the existing landscapes, our proposition can evolve along with future scientific and technological development without the need to change the formalism of the framework. This latter point is of high interest, as current technological developments foreshadow a vast array of applications for massifly-parallel, so-called "deep" sequencing technologies. The throughput and precision already achieved with these technologies make it very likely that within the next several years essentially all current genomics and RNomics methods will be sequencing-based. Additional investigations, such as the direct sequencing and quantification of for example small nuclear RNAs, also seem within reach. Our proposition to use probability landscapes for the integration of such data is – as it is inspired by and organized along the DNA sequence – a natural solution.

## Conclusion

Probability landscapes, which include as reference set the probabilistic representation of the genomic sequence, can be used to discover and analyze correlations efficiently amongst the initially heterogeneous and un-relatable descriptions and genome-wide measurements. Furthermore, this structure is usable as a support for automatically generating and testing hypotheses for alternate gene regulatory grammars and the evaluation of those through the statistical analysis of the high-dimensional correlations between the grammar to be tested, genomic sequence, sequence annotation, and experimental data. Finally, this structure provides a concrete and tangible basis for attempting to formulate a mathematical description of gene regulation in eukaryotes on a genome-wide scale. Interestingly, our propositions concerning the decomposition of genome annotation information is consistent with novel ideas concerning the understanding of the nature of genes recently published [38].

## Methods

### Constructive measures for feature probability layers

We have introduced the concept of a unified probability landscape for functional annotation of genomic sequences. Now we shall discuss how such probability layers are constructed in concrete terms. As shown, three principal types of information have to be treated. The main difference between these three types of information is not to be found in their specific nature, which is ultimately directly or indirectly derived from experimental observations, but rather, as we will see below, in the nature of the quality of estimation. Whereas the partition into three types is rigorously based on this difference, their denominations are only circumstantial and do not reflect exact boundaries. For each type we discuss how the feature probability layer is derived and how associated quality measures of the probability of feature probability can be computed.

### Genome sequence

This is the trivial case. As discussed above the ensemble of observed nucleotide sequences for a population, and later, sub-populations, is directly converted into a nucleotide frequency distribution, which is nothing but a probability distribution. Computation of the probability of feature probability is not yet state-of-the-art, but is none the less intuitive. Consider the case where *N*_*n *_observations *k*_*α*, *n *_of the nature *X *= {A, G, C, T, -} of nucleotide *n *are given by *N*_*n *_experiments labeled *α *= 1..*N*_*n*_. The estimated fraction of nucleotide *X *at position *n *is thus given by:

(1) $${\hat{P}}_{X,n}=\frac{1}{N_{n}}\sum_{\alpha =1}^{N_{n}}\delta_{X,k_{\alpha ,n}}$$

This quantity is a random variable normally distributed in the limit of *N*_*n *_going to infinity. Its mean represents the true probability of observation. Its standard deviation describes the quality, or probability density, of observing this nucleotide frequency, and is given by:

(2) $$\sigma_{X,n}=\sqrt{\frac{{\hat{P}}_{X,n}(1-{\hat{P}}_{X,n})}{N_{n}}}$$

Hence, the quality of a nucleotide probability measure in the genomic sequence scales directly and in an inverse square-root fashion with the number of independent observations at location *n*. Obviously, any new sequence information covering *n *can be used to update both the feature probability (eq. 1) and its quality (eq. 2). It is because of the high technical quality of today's different sequencing methods generating discrete observations with negligible error that we do not have to consider the technical contribution to the variance, which would be method specific.

### Sequence annotation

The type of sequence annotations is very variable, so is their quality. However, sequence annotation information is mainly based directly or indirectly on sequencing information as well. Consider for instance how gene annotations are obtained. On the one hand direct measures for expressed sequences are gathered by sequencing cDNAs and expressed sequence tags (ESTs). Such information is combined on the other hand with bioinformatical analysis of the genomic sequence such as open-reading frame mapping by translating the genomic sequence into all six possible reading frames and comparing those to known cDNA, EST and protein sequences. Other types of information that are considered in generating a gene annotation concern plausible or measured start and termination signals, plausible or measured exon-intron boundaries and so forth [30-32]. Even when considering predicted or measured secondary and tertiary protein structures, this information is ultimately derived from DNA sequence information or is superposed upon such information. Similar considerations apply to physical features of DNA such as intrinsic bend or elasticity, to telomere and centromere annotation, repeat and variable region annotation, and all other information that is today routinely gathered in sequence annotation databases [30-32]. Therefore, the same considerations as for genomic sequence apply. The main difference between genomic sequence and genome sequence annotations with respect to the feature probability layers lies in the fact that sequence annotations mostly concern sets of nucleotides rather than individual nucleotides. For example, the probability of observing an exon is not only the probability resulting from regarding a set of nucleotides jointly but is then also attributed uniformly to this entire set, creating a step, or more generally a piece-wise constant, function at the genome level. Every observable considered thereby will be used to generate an independent probability profile/layer over the genome sequence. Hence, a separate layer for each kind of sequence annotation is generated as illustrated in Figure 2.

When considering genome sequence annotations two general cases have to be distinguished in the calculation of feature probabilities. First, as in the genomic sequence, the technical variability of the underlying experimental method does not prevent discrete observables being obtained. In this case the estimated fraction of feature *x *of the nature *X *= {feature is present, feature is absent} is calculated according to (eq. 1) and its quality according to (eq. 2), where *k*_*α*,*n *_equals unity if the feature is present at genome position *n*. A feature can be any biological information or prediction that can be annotated to the genome. Second, the alternate case of continuous observables is a generalization of (eq. 1) and (eq. 2) where the methodological contribution to the variance is considered. Consider the case of *N*_*x*,*n *_observations *k*_*x*, *α*, *n *_at genome position *n *of continuous feature *x *labeled *α *= 1..*N*_*x*, *n*_. The estimated probability that feature *x *takes is a value between *k *and *k *+ Δ*k *is given by:

(3) $${\hat{P}}_{x,n}(k)\Delta k=\frac{1}{N_{x,n}}\sum_{\alpha =1}^{N_{x,n}}\chi_{\left[k,k+\Delta k\right]}(k_{x,\alpha ,n})$$

where χ denotes the step function taking value 1 inside the interval [*k, k *+ *Δ k*] and 0 elsewhere. *Δk *is an arbitrary step ideally corresponding to the resolution of the information generating method, and in practice controlled by the number *N*_*x*, *n *_required to get a good statistics for this normalized histogram (eq. 3). The probability that the summand χ_ [*k*,*k*+*Δk*]_(*k*_*x*, *α*, *n*_) equals unity is given by some value *p*_*x*,*α*,*n*_(*k*)Δ*k *including now the α-dependent methodological contribution in addition to the biological variability. The probability-density of feature probability thus remains a Gaussian for sufficiently large *N*_*x*,*n*_, fully characterized by its mean:

(4) $$\langle {\hat{P}}_{x,n}(k)\rangle =\frac{1}{N_{x,n}}\sum_{\alpha =1}^{N_{x,n}}p_{x,\alpha ,n}(k)$$

and variance:

(5) $$\begin{array}{l} \text{Var}({\hat{P}}_{x,n}(k))=\frac{1}{N_{x,n}^{2}\Delta k}\times \\ \sum_{\alpha =1}^{N_{x,n}}p_{x,\alpha ,n}(k)\left[1-p_{x,\alpha ,n}(k)\Delta k\right] \end{array}$$

The actual choice of Δ*k *will reflect the compromise between a good sampling of the distribution, small Δ*k*, see (eq. 3), and a good statistical quality, see (eq. 5).

It can easily be shown that any type of genomic sequence annotation information can be translated to feature probabilities and probability density estimates as quality measures of the feature probabilities according to these formalisms.

### Experimental data

Functional genomics experimental data are very different in nature from sequence annotations, which ultimately are also experimentally derived. Most importantly, the former are in general specific to a biological condition or state [34]. A transcriptome profile for instance is a snapshot of the transcriptional activity of a given cell in a given, and often ill-defined, cellular state. In consequence, the space of possible states that the system can adopt is not necessarily entirely defined, which is not the case for sequence information and sequence annotation information [2]. As defined above, the possible system states for sequence information are simply {A, C, T, G, -}. In the case of sequence annotation information, let us here for illustrative purposes consider the annotation of a genomic sequence with a probability of forming an α-helical protein structure, which is continuous for the interval [0,1]. The intrinsic state for this type of information is exclusively a function of the sequence itself (which is available, defined) and not the cellular state (which is not well defined). Note that obviously this probability of forming an α-helical structure can then be modulated by the cellular state (interacting factors, ionic strength and so forth). However, this is a discrete observable that would be annotated in our scheme as an independent feature probability layer resulting from experimental observation (amount of interacting factor present, etc.). Therefore, in the sequence annotation case, the boundaries of the space of possible states are defined as a function of the sequence, can be exploited in the calculation of the feature probability profile, and do not need any revision unless the sequence information is revised (see our discussion above). Functional genomics data, however, by themselves provide knowledge on neither the discrete number of possible systems states, nor the boundaries of a continuous distribution of systems states. Only a complete map of all possible cellular states using the defined technology would provide such a definition for this technology. Consider again the case of transcriptome profiles, where we measure the relative transcription activity of a large set of genes simultaneously under a single given condition. There is no way to know whether a gene *G *that has been determined to have some relative activity *A*_G,C1 _under condition *C*_1 _could not have a higher absolute activity *A*_G,C2 _under another condition *C*_2_. Only if we had complete knowledge of the gene's activity under all possible conditions could we determine its absolute maximal and minimal expression activity and use it to scale any experiment once-for-all. Since this is principally not achievable, the probability feature maps for experimental data of a given technology will have to be reevaluated entirely and subsequently rescaled with every new experimental data set. The problem is thus that the boundaries of the possible system states are unknown and have themselves to be experimentally observed. This holds although no absolute quality assessment can be obtained. For this reason the constructive methods for calculating feature probability profiles and feature probability quality profiles are different from those used to obtain the sequence probability and sequence annotation probability profiles. In effect, whereas the latter two are calculated using the frequency of observation as a basis, for functional genomic data we need to employ a Bayesian probability calculation [39].

**Figure 4:**
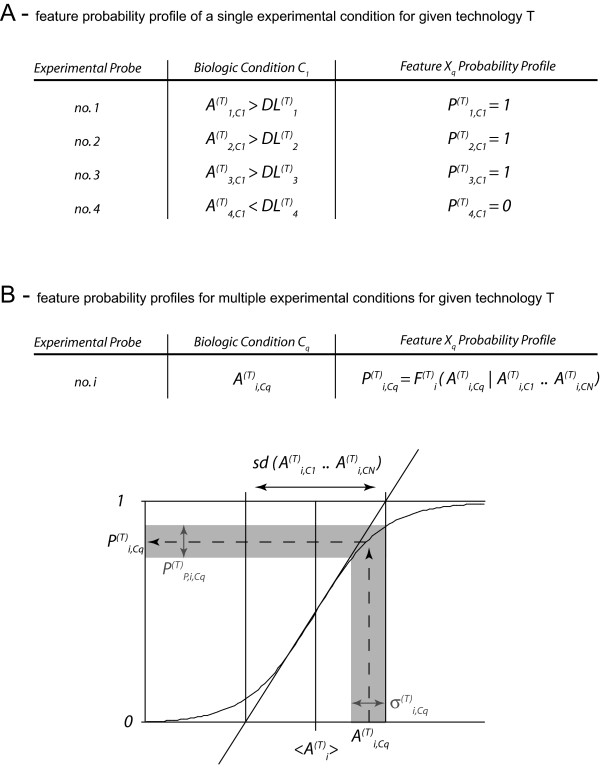
**Feature probability profile construction for experimental data**. Functional genomics experimental data are obtained associated with probes spanning and representing the entire genome at various resolutions (currently from some fifty base-pairs to hundreds of kilobases). The conversion of experimental data into probabilities requires the distinction of two cases. (**A**) In the trivial case only one experimental/biological condition has been investigated. In the absence of any additional information, the assumption that any probe returning a signal above its detection limit (*DL*) returns the maximal signal strength has to be made. Hence, any probe with a signal above its probe-specific threshold of detection will be assigned a probability of unity, whereas any probe-signal below the detection limit is set to zero. As discussed in the main text, all nucleotides covered by the probe are assigned the corresponding probability. For biological analysis this trivial case has little relevance. (**B**) For any thorough analysis several biological conditions *C*_*q *_will be investigated using the same technology *T*. As discussed, the boundaries of the possible system states are unknown. With every new experimental dataset the probability layers for each condition need to be rescaled. For this, as biological data are generally assumed to follow lognormal distributions, we use the integral over a lognormal distribution as the rescaling function *F*^(*T*)^_*i*_. Obviously, any associated signal variance stemming from technical replicates is accordingly rescaled together with the probe probability.

As outlined in Figure 4, the reevaluation and rescaling of all feature probability profiles stemming from a single technology *T *can be achieved based on the assumption that such data are always lognormally distributed by integrating over a lognormal distribution constructed from the observed weighted means, weighted variances and detection limits *DL *for each probe [40]. Calculation of the feature probability quality profile, however, becomes very complex because different technologies are often being used to measure *a priori *the same biological event, but given a large heterogeneity in both absolute and relative (between individual probes) quality return very different measures for what is supposed to be the same observable [28]. The feature probability quality profile is consequently a function of the observable itself (the probe associated signals), the biological condition, and the technology used. As the feature probability profiles are being recalculated with every new dataset stemming from the same technology, the associated feature probability quality profiles also need to be recalculated at the same time. Since the feature probability quality profiles in the case of experimental data do not only associate a quality variance estimate over the probability calculated from an existing observation and its biological variance, but directly influence the calculation of the feature probability profile, the entire process thus consists of three consecutive steps:

#### Step 1

In fact the measures for a given probe *i *obtained under different biological conditions might, and usually do, differ markedly in accuracy and faithfulness; the value *A*_*i*,*Cq *_has thus to be supplemented with a quality variance estimate *σ *^2^_*i*,*Cq*_. This variance effectively quantifies the quality of the observation, hence the value it should be given in subsequent calculations and analysis. In particular, the statistical parameters <*A*_*i*_> and *sd*_*i *_involved in the rescaling function *F*^(*T*)^_*i*_, and defined above (Figure 4B) as the mere average and standard deviation of the observations (*A*_*i*,*C*1_.. *A*_*i*,*CN*_), have to be reevaluated and turned into weighted average and weighted standard deviation:

(6) $$\langle A_{i}\rangle =\frac{1}{N}\sum_{q=1}^{N}w_{i,C_{q}}A_{i,C_{q}}$$

and:

(7) $$sd_{i}^{2}=\frac{1}{\left(N-1\right)}\sum_{q=1}^{N}w_{i,C_{q}}{\left(A_{i,C_{q}}-\langle A_{i}\rangle \right)}^{2}$$

The quality of the measurements under different biological conditions has thus a direct impact on the probability profile determination. It has also an impact on the quality assessment of the feature probability quality profile in general. Indeed, <*A*_*i*_> and *sd*_*i *_are determined within some tolerance, roughly estimated as the square root of their variance:

(8) $$\text{Var}(\langle A_{i}\rangle )=\frac{1}{N^{2}}\sum_{q=1}^{N}w_{i,C_{q}}^{2}\sigma_{i,C_{q}}^{2}$$

This shows that the natural choice for the weights is:

(9) $$w_{i,C_{q}}^{2}=\frac{const.}{\sigma_{i,C_{q}}^{2}}$$

where the constant is such that:

$$\sum_{q=1}^{N}w_{i,C_{q}}=1$$

In this way, each biological condition contributes equally to the variance of the overall statistical features; in other words, it is properly weighted relative to the other sources of information.

#### Step 2

In consequence, accounting for the quality of the different biological conditions and the uncertainty of the measurement not only updates the parameters of the rescaling function *F*_*i*_, it also replaces it with a set *F*_*i *_composed of all the possible instances of *F*_*i *_given the tolerance on <*A*_*i*_> and *sd*_*i*_. In practice, *F*_*i *_is obtained by varying the values of <*A*_*i*_> and *sd*_*i *_in an interval of width:

$$\sqrt{Var(\langle A_{i}\rangle )}\text{ and }\sqrt{Var(sd_{i})},\text{ respectively}.$$

The procedure for obtaining the variability of the probability profile estimate *P*_*i*,*Cq *_is then similar to that sketched in Figure 4B, only replacing the curve *F*_*i *_by a "fuzzy" curve *F*_*i*_.

#### Step 3

**Figure 5:**
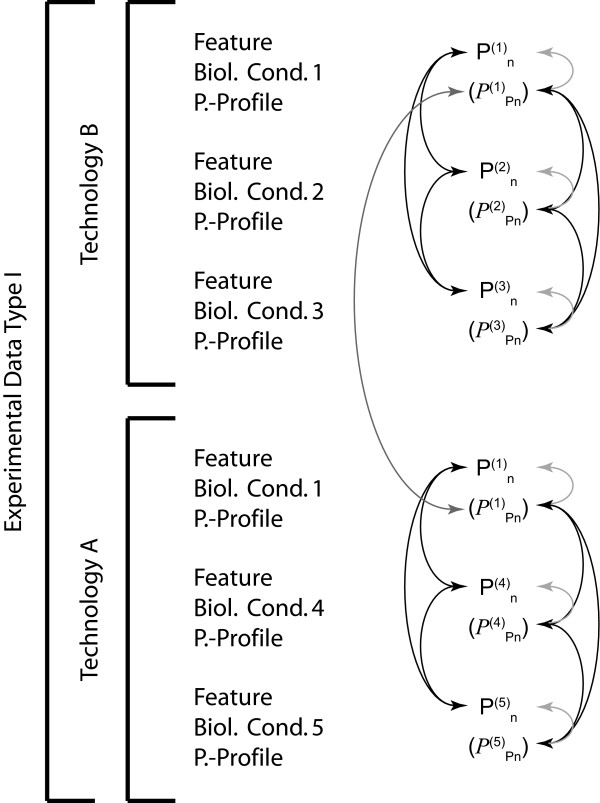
**Feature probability quality profile construction for experimental data**. The probability assigned to genomic position *n *hereby does not only depend on the measure obtained in a given experimental condition *C*_*q*_, but by the ensemble of all measures obtained for the observable located at *n *for a given technology (black arrows, left side of *P*-column). Likewise, the feature probability quality measures at *n *depend on all quality estimates (black arrows, right side of column). Those quality measures have a direct influence on the feature probability assigned to *n*, as discussed in the text (light grey arrows, right side). Finally, if data obtained under the same experimental condition are available for the same genomic location and where generated using different technologies, the *P*_*n *_values can also be weighted according to their respective quality as determined by the associated *P*_*Pn *_densities to account for technology dependent quality differences (dark grey arrow to the left). Please refer to the text for explanations.

An additional, final ingredient has to be taken into account in order to calculate the feature probability quality profiles (see Figure 5). As discussed above, very heterogeneous technologies are being used to determine the same biological observable. The heterogeneity of the technologies (e.g. different probes for the same gene, different surface and revelation chemistry, different chemical probe design, etc.) leads to a situation where every single measurement within, as well as the entire set of measurements, for an experimental condition differs in its quality from technology to technology. Furthermore, still using the example of transcriptome profiles, some technologies provide probes only for a subset of genes, and the different subsets are not necessarily identical [28]. It is for such reasons that we propose to generate independent probability feature layers for different technologies even when the experimental conditions are identical. For downstream analysis, however, in the event of overlapping information (the common subset of observables targeted with different technologies under the same experimental conditions), only the feature probability quality profile will additionally allow the information to be weighted the provided information also according to its true or experimentally perceived accuracy. In the final step of construction of the feature probability layer the feature probability quality measures have thus to be rescaled taking account of technology-specific accuracy. Note that this operation, in contrast to the other two steps only affects the feature probability quality profile and not the feature probability profile itself (Figure 5). The technology-dependent feature probability quality profile scaling function is thereby constructed using again intrinsic features of the data themselves. However, the probabilities obtained for the same observable (more precisely: its projections on the same genomic location *n*) are compared under the same experimental condition. Hence, such a quality profile rescaling is only applicable to (i) identical observables, which have been measured under (ii) identical biological conditions. Note that we have also developed a *global *rescaling and evaluation procedure for comparisons of data obtained by different technologies, which is moreover independent of the biological conditions. This type of quality assessment, however, can only be employed during downstream analysis by using statistical sequence models and hence can not be included to determine the *P*_*Pn *_values.

Briefly, as the probability measures for a given genomic location *n *for a given biological condition C_*q *_from all technologies *T *used are absolute probabilities in the interval *P*_*n *_ε [0,1], they can be directly compared even if the original signal values were completely different in nature. Furthermore, the associated probability densities *P*_*Pn*_, the quality measures, also give absolute quality assessment of the technologies for this observable, and can be used to weight each *P*_*n *_of different technological origin for the same observable.

It can readily be appreciated that the strategy we describe here can be used for any of the existing functional genomics experimental methodologies [34]. More importantly, its very general structure ensures that data generated with future technologies can also be translated into the feature probability profiles introduced here. Note that experimental data generated with technologies that provide signed measurements, such as CGH where relative over- and under-representation of genomic sequence is established at the same time, need to be split into their two relative components. Subsequently, two independent feature probability profiles are generated for the same experimental condition, one consisting of probabilities and associated probability densities for over-represented sequences; the other consisting of probabilities and associated probability densities for under-represented sequences.

## Competing interests

The authors declare that they have no competing interests.

## Authors' contributions

AL and AB have jointly investigated the mathematical, computational, and experimental aspects of the idea, initially proposed by AB, upon which this work is based. Both authors have written the manuscript together. Both authors have read and approved the final manuscript.
